# Autophagy fails to prevent glucose deprivation/glucose reintroduction-induced neuronal death due to calpain-mediated lysosomal dysfunction in cortical neurons

**DOI:** 10.1038/cddis.2017.299

**Published:** 2017-06-29

**Authors:** Cristian Gerónimo-Olvera, Teresa Montiel, Ruth Rincon-Heredia, Susana Castro-Obregón, Lourdes Massieu

**Affiliations:** 1 División de Neurociencias, Departamento de Neuropatología Molecular, Instituto de Fisiología Celular, Universidad Nacional Autónoma de México (UNAM), Ciudad de México, CP 04510, México

## Abstract

Autophagy is triggered during nutrient and energy deprivation in a variety of cells as a homeostatic response to metabolic stress. In the CNS, deficient autophagy has been implicated in neurodegenerative diseases and ischemic brain injury. However, its role in hypoglycemic damage is poorly understood and the dynamics of autophagy during the hypoglycemic and the glucose reperfusion periods, has not been fully described. In the present study, we analyzed the changes in the content of the autophagy proteins BECN1, LC3-II and p62/SQSTM1 by western blot, and autophagosome formation was followed through time-lapse experiments, during glucose deprivation (GD) and glucose reintroduction (GR) in cortical cultures. According to the results, autophagosome formation rapidly increased during GD, and was followed by an active autophagic flux early after glucose replenishment. However, cells progressively died during GR and autophagy inhibition reduced neuronal death. Neurons undergoing apoptosis during GR did not form autophagosomes, while those surviving up to late GR showed autophagosomes. Calpain activity strongly increased during GR and remained elevated during progressive neuronal death. Its activation led to the cleavage of LAMP2 resulting in lysosome membrane permeabilization (LMP) and release of cathepsin B to the cytosol. Calpain inhibition prevented LMP and increased the number of neurons containing lysosomes and autophagosomes increasing cell viability. Taken together, the present results suggest that calpain-mediated lysosome dysfunction during GR turns an adaptive autophagy response to energy stress into a defective autophagy pathway, which contributes to neuronal death. In these conditions, autophagy inhibition results in the improvement of cell survival.

Glucose supplied from the blood–brain-barrier is the main energy substrate in brain. Any decrease in blood glucose or insufficient supply to the brain results in the impairment of neuronal function. When glucose decreases below 20 mg/dl the hypoglycemic coma can occur resulting in brain injury if not opportunely reversed.^1^ Hypoglycemia is the main complication of insulin treatment in type-1 diabetes mellitus patients with a tight glycemic control. These patients frequently experience episodes of moderate hypoglycemia and are at risk to fall in the hypoglycemic coma leading to brain glucose deprivation (GD). Experimental studies show that rats exposed to the hypoglycemic coma after insulin administration, exhibit selective brain damage in vulnerable regions such as the cortex and the hippocampus, which leads to cognitive impairment.^2, 3^ Recent data from our group suggest that the early signals triggered during GD contribute to delayed hypoglycemic neuronal damage involving oxidative stress, endoplasmic reticulum stress, calpain activation, and caspases-7 and -12 increased activity.^4, 5, 6^ Other studies have suggested that oxidative stress and PARP activation following glucose reintroduction (GR), importantly contribute to delayed neuronal damage.^3, 7^

Macro-autophagy (here referred to as autophagy) is a lysosome-mediated intracellular catabolic mechanism responsible for the bulk degradation of damaged or dysfunctional cytoplasmic proteins and intracellular organelles and recycling of its components,^8^ among other functions. It is characterized by the engulfment of cellular components into double- or multiple-membrane cytoplasmic vesicles called autophagosomes that form from a membranous structure called phagophore. Autophagosomes ultimately fuse with lysosomes forming autolysosomes. Several protein complexes regulate autophagy induction, autophagosomes formation and maturation into autolysosomes. The PtdIns3K-III/BENC1 complex is necessary for autophagosome initiation; lipidation and redistribution of the cytoplasmic protein LC3 towards the phagophore contributes to its elongation around the cargo to be engulfed; and an autophagic receptor like p62/SQSTM1 allows cargo recognition. Upon autolysosome maturation, lysosomal catabolic enzymes degrade its content, including the autophagic receptor p62/SQSTM1, providing the cell of building blocks in order to maintain the energy status.^9^ When the autophagic flux is impaired, the cargo is not degraded leading to an accumulation of p62/SQSTM1.

In neurons, basal autophagy is important for the turnover of organelles and long-life proteins preventing their accumulation, which can disrupt neuronal function^10^ and lead to neurodegeneration associated with Parkinson’s, Huntington’s and Alzheimer's disease.^11, 12, 13^ In addition, autophagy constitutes a major protective mechanism that allows the cell to survive in response to multiple stressors.^14^ However, in some circumstances autophagy may lead to cell death and contribute to brain damage.^15^ Several *in vivo* models of ischemia and hypoxia/ischemia have reported autophagy activation^16, 17, 18^ and both a protective and a harmful role of autophagy have been proposed.^18, 19, 20^ To date, little is known about autophagy as an adaptive response to hypoglycemia, nor whether it contributes to hypoglycemic brain injury. Therefore, in the present study we have investigated the dynamics of autophagy in an *in vitro* model of GD and GR, and aimed to elucidate whether it contributes to neuronal injury and which are the mechanisms involved. Results show that neuronal cultures rapidly responded to GD enhancing autophagosome formation, while the autophagic flux (i.e., degradation) occurred upon glucose replenishment. Calpain activation during GR led to lysosome membrane permeabilization (LMP), impaired autophagic flux and decreased cell survival. During late GR autophagosome accumulation was observed in well-preserved cells and the number of viable neurons containing autophagosomes and lysosomes was increased when calpain was inhibited. Deficient autophagy in the present conditions contributed to neuronal death, as its inhibition by 3-MA or *Atg7* knockdown increased cell viability. Altogether, results suggest that GD induced a rapid autophagic response, which contributed to neuronal death due to the impairment of the autophagic flux and lysosomal damage mediated by calpain activation.

## Results

### There are two waves of autophagy induction during GD/GR

**Figure 1:**
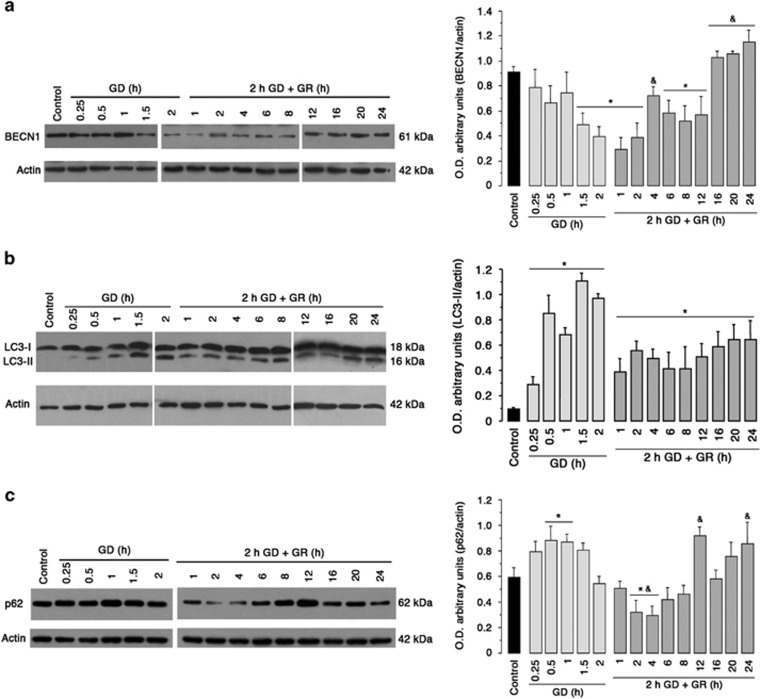
There are two waves of autophagy induction in cortical cultures exposed to GD/GR. Representative immunoblots and quantification of BECN1/actin ratio (**a**), LC3-II/actin ratio (**b**) and p62/SQSTM1/actin ratio (**c**). Bars represent mean±S.E.M. (*n*=4–5). Data were analyzed by one-way ANOVA followed by a Fisher’s *post hoc* test *P<0.05 versus control, and ^&^*P*<0.05 versus 2 h of GD

To investigate the dynamics of autophagy in response to GD/GR, proteins that function at three stages of the autophagic pathway were monitored.^21^ First, we evaluated the content of BECN1, a protein necessary for the initiation of autophagosome formation, and observed a progressive decrease during GD and early GR (1–2 h; Figure 1a). At 4 h of GR BECN1 expression was recovered and increased from 16 to 24 h after GR (Figure 1a). Subsequently, we evaluated the lipidation of LC3-I into LC3-II, which is a hallmark of autophagosome formation and p62/SQSTM1 content as indicative of autophagic flux. LC3-II and p62/SQSTM1 levels significantly increased during the 2 h GD period (Figures 1b and c). Inhibition of lysosome degradation with chloroquine (CQ) during this period did not cause a further increase in these proteins (Supplementary Figures S1A and B), suggesting the impairment of the autophagic flux, which correlated with ATP depletion (Supplementary Figure S1C). Immediately after glucose replenishment, LC3-II and p62/SQSTM1 significantly declined and CQ treatment during 2 and 4 h after GR augmented LC3-II and p62/SQSTM1 levels (Figures 1b, c, Supplementary Figures S1A and B), suggesting autophagic flux reestablishment, which correlated with a partial recovery of ATP content (Supplementary Figure S1C). Finally, at late GR (12–24 h), LC3-II and p62/SQSTM1 showed a second peak of accumulation (Figures 1b and c). In agreement with these results, cortical cultures exposed to 1 h GD showed intense LC3 immunoreactive puncta, and autophagosome-like structures identified as double or multimembrane vesicles, were detected by electron microscopy. These structures were not detected in control cells (Supplementary Figures S2A and B).

**Figure 2:**
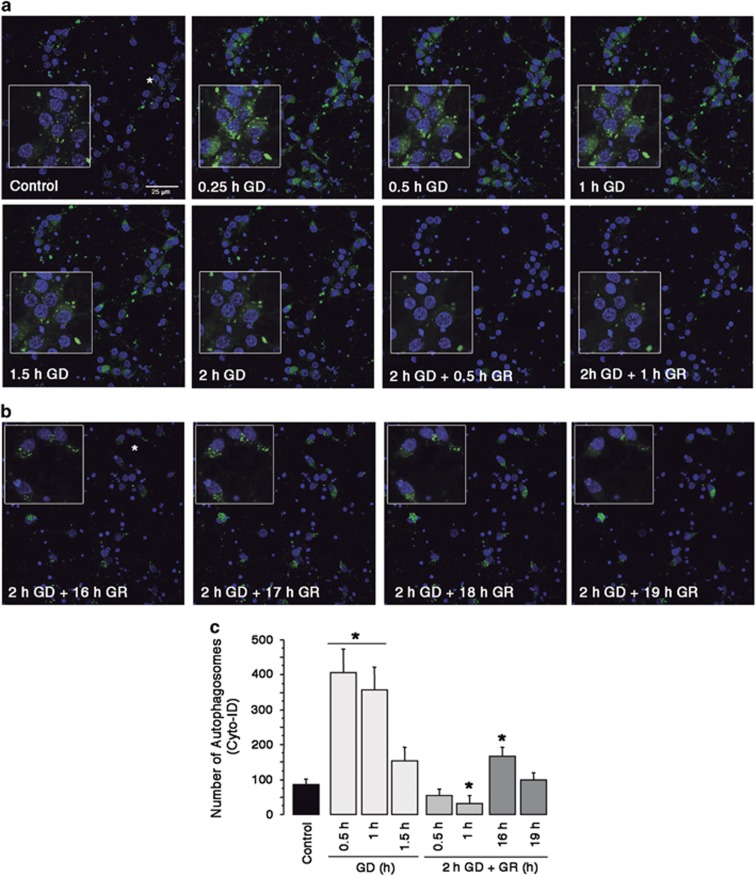
Autophagosomes are formed in cortical cultures during GD and late GR. Representative images of time-lapse autophagosome formation in cortical cultures exposed to GD/GR using the Cyto-ID detection kit (green) and Hoechst counterstaining (blue) (**a** and **b**). Asterisks indicate magnification zones. Graph shows the number of autophagosomes at different times after GD/GR (**c**). Bars represent mean±S.E.M. (*n*=3–6). Data were analyzed by one-way ANOVA followed by a Fisher’s *post hoc* test *P<0.05 versus control

Then we aimed to follow the time-course of autophagosome formation and degradation during the GD and GR periods. Time-lapse live confocal imaging was performed using Cyto-ID, a green fluorochrome that labels autophagosomes.^22^ After 15–30 min of GD autophagosomes appeared in many cortical neurons while in control cultures there were only a few. Labeled autophagosomes remained visible after 1 h GD, while soon after GR (0.5–1 h) they almost completely disappeared (Figures 2a and c), suggesting autolysosomal maduration. After 16 h of GR many autophagosomes were observed in well preserved cells that contained large nuclei and they declined after 3 h (Figures 2b and c). These results are consistent with the autophagy proteins abundance observed by immunoblot, and suggest that GD induced a rapid accumulation of autophagosomes due to deficient autophagic flux, which is reestablished after glucose replenishment. During late GR, autophagosomes are formed again in well-preserved neurons suggesting that autophagy is reactivated.

### GR induces delayed neuronal death

**Figure 3:**
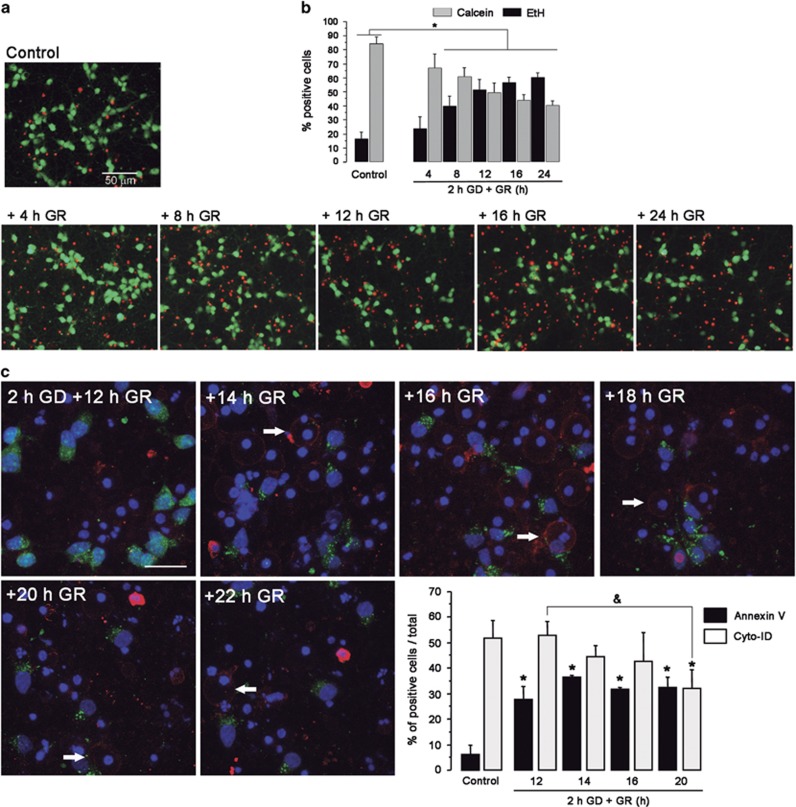
Neurons progressively die during GR and apoptotic cells are devoid of autophagosomes. Representative images and quantification of live/dead neurons using EtH (red) and Calcein-AM (green) (**a** and **b**); and Cyto-ID (green), Annexin V (red; arrows) and Hoechst (blue) during different times of GR after exposing cells to 2 h GD (**c**). Graphs show the percent of cells positive to EtH or Calcein-AM (**b**) and to Annexin V or Cyto-ID (**c**) at different times after GR. Bars represent mean±S.E.M. (*n*=3–4). Data were analyzed by one-way ANOVA followed by a Fisher’s *post hoc* test *P<0.05 versus control, ^&^*P*<0.05

According to the above-described results autophagosomes are present in cells surviving late GR. A time-course of cell death throughout GD/GR was followed using the live/dead assay (calcein-AM/Ethidium homodimer (EtH). Cells exposed to GD alone and analyzed immediately after, did not show any change in viability (not shown). However, when cultures underwent 2 h of GD and GR for different times, they showed a progressive increase in the number of dead neurons (red cells) accompanied by a decrease in cells that were alive (green cells). These changes were statistically significant from 8–24 h GR suggesting that the execution of cell death progressively occurred during this period, leading to a 60% reduction of viable neurons at 24 h (Figures 3a and b). To evaluate whether apoptosis was involved in cell death time-lapse experiments were performed using Annexin V, a common marker for apoptosis that detects the externalization of phosphatidylserine and Cyto-ID to monitor autophagosome formation. It was observed that neurons positive to Annexin V (red cells) appeared at 12 h after GR and its number remained constant until 20 h, while the number of autophagosome-containing neurons significantly decreased at 20 h (Figure 3c). Cells positive to Annexin V showed condensed nuclei and no autophagosomes suggesting that autophagy is not undergoing in cells experiencing apoptosis. Cell survival was increased by incubation with the caspases inhibitors QVD-OPH (pan-caspase inhibitor) and Z-DEVD-FMK (caspase-3/7 inhibitor) during GR (Supplementary Figure S3A), further supporting that neuronal death is executed during this period. In agreement, a significant increase in the number of TUNEL-positive nuclei was observed after GD/GR, which was reduced by QVD-OPH (Supplementary Figure S3B).

### Autophagy is involved in neuronal death induced by GD/GR

**Figure 4:**
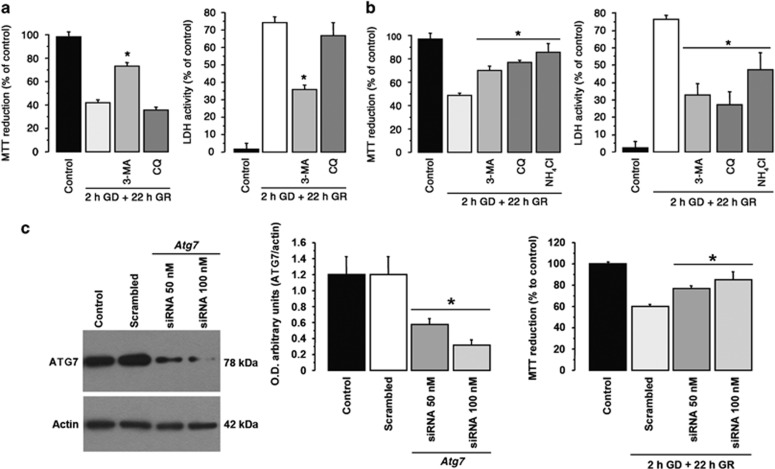
Autophagy inhibition reduced GD/GR-induced neuronal death. MTT reduction and LDH activity of cortical cultures exposed to GD/GR. The indicated inhibitors were incubated during GD (**a**) or immediately after GR (**b**). Representative immunoblots of ATG7 protein levels in control cultures and cultures incubated with scrambled siRNA or siRNA targeting *Atg7* (left graph); MTT reduction in control cultures, with100 nm scrambled siRNA 50 nm or 100 nm siRNA against *Atg7* is shown in the right graph (**c**). Bars represent mean±S.E.M. (*n*=4–10, **a** and **b**, and *n*=3–7, **c**). Data were analyzed by one way ANOVA followed by a Fisher’s *post hoc* test *P<0.05 versus GD (**a** and **b**) or versus scrambled siRNA (**c**). 3-MA, CQ, NH_4_Cl

The experiments described above suggest that cells surviving delayed GR underwent autophagy, while apoptotic cells did not form autophagosomes. Thus, we aimed to test whether inhibition of autophagosome formation would reduce cell survival. However, reduction of autophagosome formation by 3-MA, protected neurons from death when it was incubated either during GD or GR (Figures 4a, b, Supplementary Figures S4A and B). In agreement, cultures treated with 3-MA during GR showed less TUNEL-positive cells (Supplementary Figure S3B). To confirm this result, we tested neuronal viability when *Atg7* expression was silenced using RNAi. As shown in Figure 4c, *Atg7* RNAi significantly reduced the expression of ATG7 and augmented cell viability. These results indicate that inhibition of autophagy prevented neuronal death. We also tested whether autophagic degradation is involved in neuronal damage using CQ or NH_4_Cl during GR. Both compounds increased cell survival (Figure 4b). These results suggest that autophagy induction during GD/GR failed to prevent neuronal death. However, cells surviving late GR were capable to reactivate autophagy.

### Calpain activated during GR mediates autophagic/lysosomal dysfunction contributing to neuronal death

**Figure 5:**
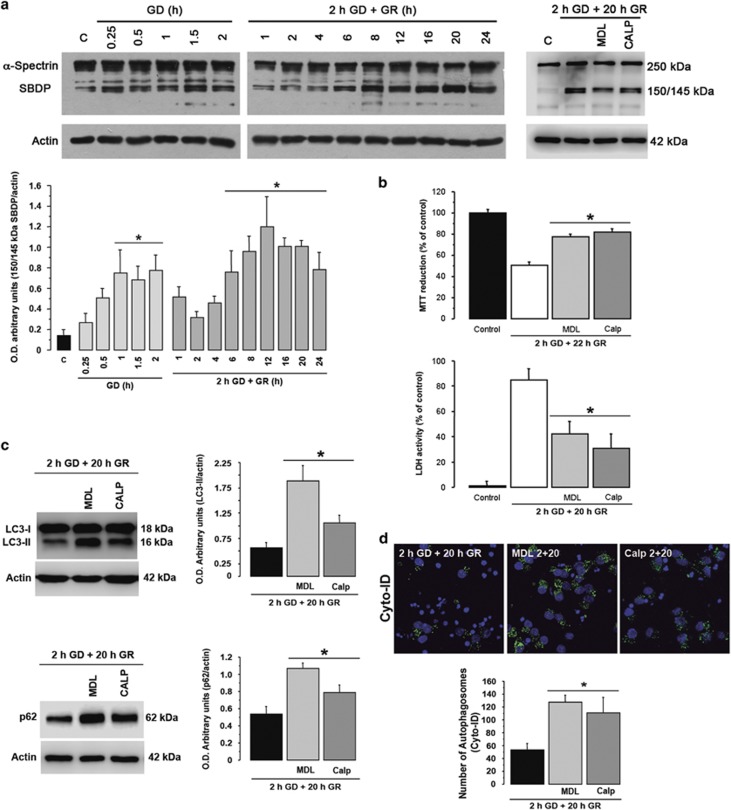
Calpain is activated in cortical cultures exposed to GD/GR. Representative immunoblots and quantification of the calpain cleaved 150/145 kDa spectrin fragments at different times of GD and GR, and effect of calpain inhibitors (**a**). MTT reduction and LDH activity of cultures exposed to GD/GR with or without calpain inhibitors. MDL or Calpastatin were added during GR (**b**). Representative immunoblots and quantification LC3-II/actin and p62/SQSTM1/actin levels in cultures exposed to GD/GR with or without calpain inhibitors (**c**). Representative images and quantification of the number of autophagosomes in cultures exposed to GD/GR with or without calpain inhibitors (**d**). Bars represent mean±S.E.M. (*n*=4 **a**; *n*=4–10 **b**; *n*=6–7 **c**, *n*=3–4 **d**). Data were analyzed by one way ANOVA followed Fisher’s *post hoc* test *P<0.05 versus control or GD

Calpains are calcium-dependent proteases that have been identified as molecular links between autophagy and apoptosis.^23, 24^ Also, we have reported that calpain is activated during GD and contributes to neuronal death in cultured hippocampal neurons.^5, 6^ To test whether calpain contributes to autophagy impairment and the subsequent death of neurons, a time-course of calpain activity was followed. The processing of *α*-spectrin into 150–145 kDa breakdown products (SBDP), produced by calpain, was monitored by immunoblot. Calpain products significantly increased during GD and late during GR (6–24 h; Figure 5a). Then, we tested whether calpain inhibition by MDL or Calpastatin, an endogenous inhibitor of calpain, prevented neuronal death. As expected, inhibition of calpain by MDL or Calpastatin reduced the production of the 150–145 kDa (Figure 5a), and increased cell survival when present during GR, as detected by the MTT, lactate dehydrogenase (LDH) activity and TUNEL assays (Figure 5b and Supplementary Figure S3B), suggesting that calpain activation contributes to the execution of delayed neuronal death.

Then, we aimed to analyze whether calpain activity inhibits the autophagic pathway. Calpain inhibition by MDL or Calpastatin during GR significantly increased autophagic markers (LC3-II and p62/SQSTM1) as well as the number of autophagosomes at 20 h after GR (Figures 5c and d). These results support that calpain activity impaired autophagy and that its inhibition increased the number of viable cells undergoing autophagy.

**Figure 6:**
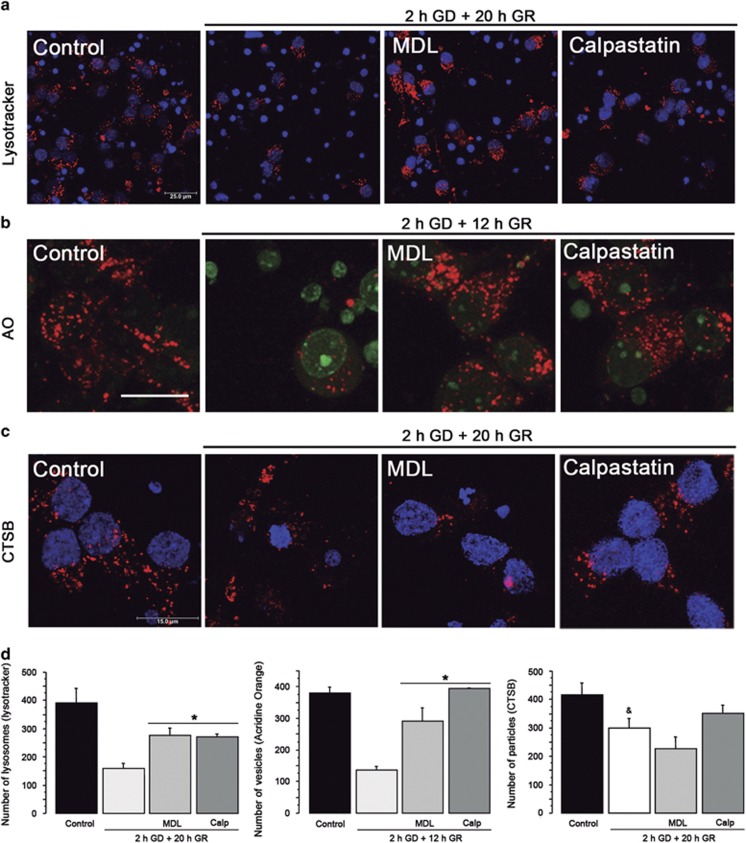
Calpain inhibition increases lysosomes, AO-positive vesicles and CTSB immunoreactive particles at late GR. Images of representative experiments showing, Lysotracker (red) (**a**), Acridine Orange (**b**) and CTSB (red) (**c**) and Hoechst counterstaining (blue). MDL and Calpastatin were added at the onset of GR. Scale bar=25 *μ*m (**a**), 15 *μ*m (**b** and **c**). Graphs show the total number of lysosomes, AO vesicles and CTSB immunoreactive particles in the different experimental conditions (**d**). Bars represent mean±S.E.M. (*n*=3). Data were analyzed by one way ANOVA followed by a Fisher’s *post hoc* test *P<0.05 versus GD, ^&^*P*<0.05 versus control

Activated calpain can cleave proteins of the lysosomal membrane inducing LMP and thereby cathepsin release and cell degradation.^25, 26^ Thus, we investigated this possibility monitoring lysosome activity and integrity using Lysotracker and Acridine Orange (AO). As shown in Figure 6a, control neurons showed many intense fluorescent Lysotracker positive-vesicles (lysosomes and autolysosomes), while the number of total lysosomes was reduced after 20 h of GR (Figures 6a and d). Calpain inhibitors significantly increased the number of well-preserved neurons and the total number of lysosomes at 20 h after GR (Figures 6a and d). In agreement, control cells showed many acidic vacuoles sequestering AO and non-fluorescent nuclei (lacking AO), while in cells exposed to 2 h GD and 12 GR, the number of orange vesicles was significantly reduced and nuclei appeared intensively stained in green (AO bound to DNA). These observations suggest LMP and thereby the leak of AO from lysosomes, allowing its translocation to nuclei. Conversely, in cells treated with calpain inhibitors, the number of acid vacuoles in the cytosol was restored, while nuclei staining was substantially reduced (Figures 6b and d). These results support that calpain activation during GR induces LMP, altering lysosome integrity, which is prevented by MDL and Calpastatin.

To further corroborate that LMP occurs during GR, we analyzed by immunostaining whether cathepsin B (CTSB) would display a cytoplasmic distribution. As shown in Figure 6c, control cultures showed immunoreactive puncta to CTSB in the soma of many cells, suggesting its localization into lysosomes. When cultures were exposed to GD/GR, the number of CTSB immunoreactive particles was significantly reduced and diffuse immunoreactivity appeared in the cytosol of cells showing condensed nuclei (Figure 6c). In accordance, we observed a decrease in CTSB active fragment in the lysosomal enriched fraction after GR and an increase in the cytosol suggesting its leakage from the lysosome (Supplementary Figure S5A). This result is in agreement with the reduced staining of lysosomes observed with lysotracker and AO. Accordingly, the number of cells showing immunoreactivity to the lysosomal protein LAMP1 was significantly reduced in cultures exposed to GD/GR (Supplementary Figure S5B).

The data described above support the hypothesis that cathepsins may participate in the cell death cascade after LMP. Therefore, the specific cathepsin inhibitiors, pepstastin A (CTSD) and CA074 (CTSB), were tested against neuronal death. As shown in Supplementary Figure S5C cathepsins inhibition resulted in increased neuronal survival and a significant reduction in the number of TUNEL-positive cells (Supplementary Figure S3C), suggesting cathepsins participate in neuronal damage. These data also suggest a correlation between lysosomal loss and neuronal death. In agreement, cells incubated in the presence of Calpastatin recovered the control CTSB immunoreactive pattern (Figure 6c), increased the number of cells containing LAMP1 and prevented CTSB release from lysosome (Supplementary Figures S5A and C). MDL also induced the recovery of LAMP1 immunoreactivity and prevented CTSB release but the number of CTSB-positive particles remained low, suggesting unspecific CSTB inhibition by MDL.

**Figure 7:**
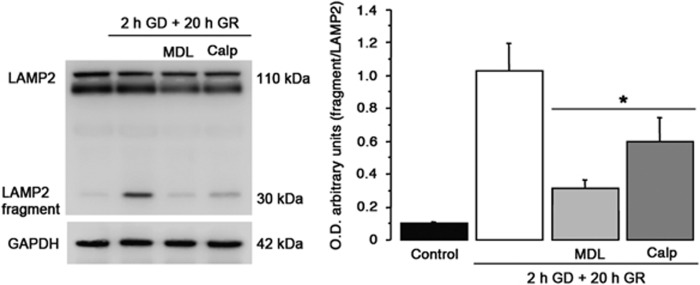
The lysosomal membrane permeabilization induced during GR is inhibited by calpain inhibitors. Representative immunoblot showing the cleavage of LAMP2 after 20 h of GR into a 30 KDa fragment. LAMP2 cleavage was inhibited by calpain inhibitors. MDL and Calpastatin were added at the onset of GR. Bars represent mean±S.E.M. (*n*=5). Data were analyzed by One way ANOVA followed by a Fisher’s *post hoc* test *P<0.05 versus control

To investigate the mechanism associated with LMP induced by GD/GR, the calpain-mediated cleavage of the lysosomal protein LAMP2 was tested by immunoblot in cells exposed to 2 h GD and 20 h GR. As shown in Figure 7a, 30 KDa product of LAMP2 was observed after 20 h of GR. LAMP2 cleavage into this fragment was prevented in cells incubated in the presence of MDL and Calpastatin. These results confirmed that LMP was induced during GR and that it was dependent on calpain activity.

## Discussion

Adaptive autophagy in response to acute stress, including energy stress, contributes to restore physiological conditions and exerts a cytoprotective effect. However, autophagy is vulnerable to other stress signals that can disrupt its function. Brain GD leads to energy stress and neuronal death but the precise molecular mechanisms involved in hypoglycemic brain damage are not completely understood and a whole picture of the events taking place throughout the hypoglycemic and the glucose reperfusion periods is still lacking. Hence, we have followed the dynamics of the autophagic pathway throughout the GD and GR periods in cortical cultured neurons and dissected the mechanism involved in the failure of the neuroprotective effect of autophagy in response to GD-generated energy stress.

We provide morphological and biochemical evidence supporting that autophagosomes are accumulated during the GD period, while the autophagic flux is active upon GR. This leads to the degradation of autolysosomes and their content as evidenced by the decrease in LC3-II and p62/SQSTM1. Previous reports have shown in hippocampal and cerebellar granule neurons that excitotoxic insults block the autophagic flux leading to the accumulation of autophagosomes, which is detrimental for cell survival.^27, 28^ We demonstrated that the early accumulation of autophagosomes during GD results from a deficient autophagic flux possibly due to decreased lysosome activity resulting from ATP depletion. The activity of the lysosomal proton pump is ATP-dependent and ATP deficiency inhibits lysosomal activity impairing the autophagosome-lysosome fusion.^29^ In agreement, we have previously reported that the ketone body, beta-hydroxybutyrate added as an alternative fuel, preserved ATP levels stimulating the autophagic flux and reducing neuronal death.^30^

An important finding of the present study is the demonstration that neuronal death induced by GD/GR, occurs progressively during the late stages of GR. Surviving cells, not positive to Annexin V, contained autophagosomes suggesting they activate autophagy as a pro-survival signal. These results contrast with the observation that autophagy contributes to the damage of neurons, as 3-MA and *Atg7* down regulation, conferred protection against neuronal death. Similarly, late autophagy inhibition by CQ and NH_4_Cl preserved cell survival. These observations indicated that reducing the autophagosome overload during GD and the subsequent excessive autophagic degradation during GR, improved neuronal viability. 3-MA not only reduced the number of cells containing autophagosomes but also the number of autolysosomes (Supplementary Figure S4), reducing lysosomal calpain targets and hence preventing the subsequent LMP. These observations agree with recently published data suggesting that autophagy inhibition reduced LMP due to increased Hsp70 expression and thereby preventing apoptosis.^31^

Altogether, our results suggest that autophagy is initially triggered in most of the cells as a survival response to GD. Subsequently, during the first hours of GR the autophagic flux is restored and it is followed by calpain activation. Calpain-mediated degradation of lysosomal membrane proteins, such as LAMP2, results in LMP, leading to the release of lysosome content and neuronal death. The cells surviving damage could be those capable to cope with calpain-mediated disruption of autophagy, as they show many autophagosomes, autolysosomes/lysosomes and CSTB immunoreactive particles. According to the present results, apoptosis is involved at least in part in the execution of neuronal death since Annexin V- and TUNEL-positive cells were observed late after GR and caspases inhibitors increased neuronal survival. More experiments are needed in order to elucidate the apoptotic cascade triggered in the present experimental conditions.

A second important observation is that inhibition of calpain during GR increases the accumulation of autophagosomes and autolysosomes/or lysosomes enabling cells to survive. This finding suggests that calpain activation may turn an autophagic survival response into mal-adaptive autophagy, which contributes to neuronal death. Calpains activate during ischemia and contribute to the death of neurons.^32^ We have previously reported that calpain activation mediates GD-induced neuronal damage in hippocampal cultures.^5^ Calpain activity can lead to the cleavage of certain proteins at the lysosome membrane, such as Hsp70.1 (ref. 33), LAMP2 (ref. 34) and subunit b2 of v-ATPase,^35^ leading to LMP. This causes the release of lysosome content towards the cytosol and the degradation of cell constituents. We demonstrated that late after GR there is a reduction in the number of lysosomes, AO-acidic vesicles and CTSB immunoreactive particles suggesting LMP. LMP was corroborated by the cleavage of LAMP2 and CTSB release observed during GR, which was reduced by calpain inhibitors. This finding together with the observed preservation of the number of lysosomes and AO-positive vesicles when calpain was inhibited, suggests that calpain contributed to LMP. In addition, LMP is involved in neuronal death since calpain inhibitors effectively increased cell survival. The protective effect of MDL can be attributed in part to CTSB inhibition. However, Calpastatin, which has no cathepsin inhibitory activity, also efficiently prevented neuronal death, suggesting that LMP is directly involved in cell death. These observations lead us to conclude that activated calpain during GR causes lysosome destabilization.

Cathepsins are lysosomal proteases involved in autophagic degradation and also major effectors of LMP leading to apoptosis,^25^ hence they participate in the crosstalk between autophagy and apoptosis. Pioneer studies showed an increase and re-localization of CTSB immunoreactivity towards the cytosol in post-ischemic neurons,^36, 37^ and CTSB release from lysosomes after calpain-mediated LMP, has been proposed as the main causative factor of ischemic neuronal death.^26^ Here, we show that inhibition of CTSB and CTSD protected neurons against GD/GR-induced neuronal death and that the recovery of lysosomes and CSTB immunoreactive particles correlated with increased cell survival, suggesting that extralysosome activated cathepsins might contribute to neuronal damage. It has been observed that after LMP, CTSB induces the cleavage of Bid, the release of cytochrome *C* and caspase activation.^35^ Moreover, CTSB can translocate to the nucleus and induce nuclear damage and chromatin condensation.^38^ Any of those mechanisms could happen during neuronal death. However, more experiments are needed to investigate the mechanism by which cathepsins activity might contribute to cell death in the present conditions.

In conclusion, results from the present study suggest that autophagy is early activated as a survival response to energy stress, but the activation of calpain during GR promotes LMP and CTSB release leading to deficient autophagy and apoptosis. Surviving neurons undergo autophagy and calpain inhibition enhances autophagy promoting cell survival. Hence, the present work demonstrates for the first time that the adaptive response of autophagy induced by GD/GR in cultured cortical neurons is vulnerable to calpain activity, failing to prevent neuronal damage.

## Materials and methods

### Glucose deprivation in cortical neurons

Primary neuronal cultures were prepared from rat E17 embryos as described by Brewer *et al.*^39^ All efforts were made to optimize the number of animals used and minimize their suffering. Animals were handled according to the National Institute of Health Guide for the Care and Use of Laboratory Animals (NIH publications N0. 80–23, revised 1996) with the approval of the Animal Care Committee (CICUAL, LMT01-14) of the Instituto de Fisiología Celular, UNAM. In brief, cerebral cortex was dissected and chopped, then incubated with 0.25% trypsin/10% EDTA solution at 37 °C for 3 min. The digestion was stopped with a solution containing soybean trypsin inhibitor and DNAse (0.52 and 0.08% respectively). Cells were suspended in Neurobasal Medium (Gibco, 21103-049, Grand Island, NY, USA) supplemented with 2% of B27 (Gibco, 17504-044), 0.5 mm l-glutamine, 20 *μ*g/ml gentamicine (Gibco, 15710-064) and plated at a density of 2.2 × 10^5^ cells/cm^2^ in plates pre-coated with poli-l-lysine (Sigma-Aldrich, P-1524, St. Louis MO, USA). Cells were cultured for 8 DIV at 37 °C in a humidified 5% CO_2_/95% air atmosphere. Cytosine-d-arabinoside (0.54 *μ*m) was added to cultures 4 days after plating. Experiments were carried out at 8 DIV. To induce GD, culture medium was removed and changed for DMEM free-glucose medium (Gibco, 11966-025) for different periods of time. After the GD period the free-glucose medium was replaced with the medium where cells were originally cultured (glucose reperfusion period, GR) for different periods of time.

### Cell treatments

To determine the role of autophagy in GD-induced damage, we used several inhibitors related to the autophagic pathway. Cells were treated with 3-MA 10 mm (PtdIns3K-III inhibitor; Sigma-Aldrich, M9281), Pepstatin A 2 *μ*m (Cathepsin D inhibitor; Sigma-Aldrich, P4265), CA074 50 *μ*m (Cathepsin B inhibitor; Sigma-Aldrich, C5732), CQ 20 *μ*m (Sigma-Aldrich, C6628), NH_4_Cl 20 *μ*m, MDL 20 *μ*m (Calpain inhibitor; Biomol international, Pennsylvania, USA), Calpastatin 1 *μ*m (Calpain inhibitor; Tocris 2950, Bristol, UK), QVD-OPH 20 *μ*m (Pan-caspase inhibitor; MP Biomedicals 03OPH109, Santa Ana, CA, USA) and Z-DEVD-FMK 20 *μ*m (Caspase 3/7 inhibitor, Sigma-Aldrich, C0605). These inhibitors were added either during GD or GR as described in the Results section.

### Immunoblotting

Cells were cultured in 35 mm dishes and exposed to different time periods of GD and GR. After treatment cells were washed with ice-cold PBS 0.1 m and lysed with a buffer containing (Tris-HCl pH 8.0 50 mm, NaCl 150 mm, Triton X-100 1%, sodium deoxycholate 0.5% and SDS 1%) and 2 mg/ml of protease inhibitor cocktail (Roche complete, 11626200, Indianapolis, IN, USA). Samples were centrifuged at 5000 r.p.m. at 4 °C for 5 min. Protein concentration was determined by the Lowry assay and 30 *μ*g of protein from each sample was separated in SDS-PAGE and subsequently transferred to PVDF membranes. The membranes were incubated with specific antibodies against the different autophagic markers: LC3 (MBL international, PD014, Woburn, MA, USA) 1:1000, BECN1 (Sigma-Aldrich, PRS3613) 1:1000 and SQSTM1/p62 (Cell signaling technology, 51146, Danvers, MA, USA) 1:500, CTSB (Santa Cruz Biotechnology, sc-6490-R, Dallas, TX, USA) 1:250, *α*-Spectrin (Chemicon Millipore, MAB1622, Temecula, CA, USA) 1:3500 and LAMP2 (Sigma-Aldrich, L0668) 1:1000. The reactions of primary antibodies were detected using the respective horseradish peroxidase, goat anti-mouse (Jackson Immunoresearch Laboratories, 115035-062, West Grove, PA, USA) or goat anti-rabbit secondary antibody (Jackson Immunoresearch Laboratories, 115035-003) and immunoreactivity was detected by chemiluminescent HRP substrate (Millipore Corporation, P90720, Billerica, MA, USA). Actin (1:7000; Merck Millipore, MAB1501, Temecula, CA, USA) was used as a loading control.

### Immunocitochemistry

Cells were cultured on cover slips and exposed to GD/GR. After the treatment cells were washed with ice-cold PBS 0.1 M and fixed with methanol for 20 min on ice. Cells were blocked with PBS-Albumin 5% Triton X-100 0.1% for 1 h at room temperature. Primary antibodies LC3 (1:500, MBL PD014), CTSB (1:250, Santa Cruz Biotechnology, sc-6490-R) and LAMP1 (1:250, Sigma-Aldrich, Ab1418) were incubated overnight at 4 °C and were detected using FITC (Zymed, 62-6111) or Alexa 488 (Jackson Immunoresearch Laboratories, 111-545-144) anti-rabbit antibodies, at room temperature for 2 h. Cells nuclei were stained with Hoechst 0.001% (Sigma-Aldrich, 33258) in PBS immediately after immunostaining and covered with Fluoromount-G (Electron Microscopy Sciences 17984). Images were obtained using confocal microscopy (FV 1000; Olympus) motorized FV10ASW 2.1, with Ar-488 laser (for FITC) and UV-405 nm (for Hoechst) or Leica TCS SP5 using 100 × oil immersion objective with 405 nm laser for Hoechst and 596 nm for Alexa. The number of cathepsin-positive particles was determined as described previously.^30^

### Electron microscopy

Cells were cultured in 60 mm dishes and exposed to GD. After treatment cells were washed with ice-cold Lockey’s buffer containing (NaCl 154 mm, KCl 5.6 mm, NaHCO_3_ 3.6 mm, CaCl_2_ 2.6 mm and HEPES 5 mm) and fixed with 3% of glutaraldehyde in Lockey’s buffer. Cells were post-fixed in 1% osmium tetroxide in PBS, dehydrated in graded alcohols, embedded in Epon 812, sectioned with an ultramicrotome and stained with uranyl acetate and lead citrate. Electron microscope images were taken with a Jeol 1200EX II.

### Live imaging of autophagosome formation

Cells cultured in 35 mm dishes were exposed for 2 h to GD followed by different times of GR and incubated with Cyto-ID (autophagy detection kit, Enzo Life Sciences, 51031-K200, Farmingdale, NY, USA), a green fluorochrome that labels autophagosomes and not autolysosomes or lysosomes, as the green fluorescence is labile in an acidic environment.^22^ Before the onset of GD, Cyto-ID was incubated for 20 min in culture medium. Neurobasal medium was washed using a reperfusion chamber and progressively substituted with DMEM free-glucose medium; after 2 h of GD, DMEM was changed again for Neurobasal glucose containing medium. Confocal images (Leica TCS SP5 using 63 × water immersion objective with UV-405 nm laser for Hoechst and Arg-488 nm for CytoID) were taken at the onset of GD and at different times after GD and GR. Hoechst was used as a nuclear counterstain. The number of autophagosomes was determined as described previously.^30^

### Cell viability

#### MTT cell viability assay

The viability of cortical neurons was measured by the 3-(4, 5-dimethylthiazol-2-yl)-2, 5-diphenyltetrazolium bromide (MTT Sigma-Aldrich, M2128) reduction assay, which is indicative of viable mitochondrial.^40^ Cells were exposed for 2 h to GD and 22 h of GR; after 22 h of GR MTT (60 *μ*g/ml) was added and incubated for 1 h at 37 °C. The resulting formazan salt was dissolved with 2-propanol-HCl and monitored at 570 nm in a spectrophotometer. Data are expressed as percent of control.

#### Lactate dehydrogenase (LDH) activity

Cell viability was also measured by the LDH assay. Cells were exposed to 2 h GD and 15 h after GR, 200 *μ*l of culture medium were collected for LDH activity determination. Samples were incubated with NADH 9.4 mm in K_2_HPO_4_/KH_2_PO_4_ 1 mm buffer during 5 min at room temperature. The reaction was started with pyruvate 20 mm and was followed for 5 min by the decrease in NADH fluorescence at 340 nm using a Beckman Coulter life science UV/Vis Spectrophotometer. Data were normalized to control values and are expressed as percent activity in the medium relative to the control. Cell viability was also determined by the Live/Death kit as described.^30^

### Monitoring of Annexin V positive cells

Cells were cultured on cover slips and exposed to GD/GR. After treatment Annexin V (Life technologies, Alexa Fluor 594, Eugene, OR, USA) and Cyto-ID were incubated for 20 min at 37 °C. Confocal images (Leica TCS SP5 using 63 × water immersion objective with UV-405 nm laser for Hoechst, Arg-488 nm for CytoID and He/Ne-543 for Annexin V) were taken after 20 h of GD. Hoechst was used as a nuclear counterstain

### *Atg7* silencing

At 4 DIV cells plated on 12-well plates were treated for *Atg7* RNAi silencing. Cultured medium was replaced for the Accell RNAi delivery media (GE Healthcare Dharmacon Inc.) supplemented with 2% of B27 containing 50 or 100 nm of Smart pool *Atg7* RNAi or 100 nm scrambled RNAi. After 8 h of incubation Accell medium was changed for the medium where cells were originally cultured. Cells were maintained for 8 DIV under the conditions previously described.

### ATP determination

Cells were plated on 12-well plates during 8 DIV. ATP concentration was determined immediately after 1.5 h GD or 1, 2 and 3 h after GR. ATP levels were determined by means of a luminometer through the luceferin-luciferase Chemiluminescent kit (Molecular Probes, A22066, Eugene, OR, USA), as previously described^4^ and ATP concentrations were calculated from readings obtained from an ATP standard curve (from 6.5 to 250 pmol). Protein concentration was determined by the Lowry’s method and data are expressed as pmol/*μ*g of protein.

### Lysotracker staining

Cells were cultured on cover slips and exposed to GD/GR. After treatment Lysotracker dye (Life technologies, DND-99) was incubated for 20 min at 37 °C. Confocal images (Leica TCS SP5 using 63 × water immersion objective with UV-405 nm laser for Hoechst and He/Ne-543 for Lysotracker) were taken after 20 h of GD. Hoechst was used as a nuclear counterstain. The count of Lysotracker-positive vesicles was performed by FIJI image analysis software,^41^ considering three independent experiments with three repeated technical samples. We analyzed the 10 last images of the total stack to obtain the maximum projection and gauged the parameters of ‘analyze particles’ plug-in as follows: size (area) from 0.2 to 25 *μ*m^2^ and circularity from 0.1 to 1.0 for identification of positive vesicles.

### Lysosome membrane integrity assay (Acridine Orange, AO)

Cells cultured in 35 mm petri dishes were exposed to 2 h GD and 12 h GR. After treatment cells were incubated with 2 *μ*g/ml of acridine orange for 10 min at 37 °C and washed with Hank’s balanced salt solution. Acridine orange is a fluorescent cationic dye that enters into acidic compartments (i.e., lysosomes and/or autolysosomes) where it is protonated and sequestered. Under low pH conditions, the dye emits orange/red fluorescence. However, under LMP conditions acridine orange is released and interacts with nucleic acids emitting a green fluorescence. Confocal images were taken with Leica TCS SP5 using 63 × water immersion objective Arg 458/650 nm (red; acidic vacuoles) and Arg 488/525 nm (green; nuclei). The number of acridine orange-positive particles was determined as described for lysosomes.

### Subcellular fractionation (lysosome enriched fraction)

Cells cultured in 10 mm petri dishes were exposed to 2 h GD and 12 h GR. After treatment cells were washed with ice-cold PBS 0.1 m and harvested with ice-cold PBS 0.1 m containing 2 mg/ml of protease inhibitor cocktail. Samples were centrifuged for 10 min, 1000 g at 4 °C. Pellet was homogenized using 15 strokes of the pestle of a tight fitting Dounce homogenizer in sucrose 0.32 m containing 2 mg/ml of protease inhibitor cocktail. Homogenates were centrifuged at 1000 g for 5 min to pellet the nuclei. The supernatant was centrifuged at 3000 g for 10 min. Then, the supernatant was centrifuged at 17 000 g for 15 min to pellet the lysosome enriched fraction. The supernatant was centrifuged at 60 000 g for 30 min to completely clean the cytosolic fraction.

### Statistics

All data are expressed as Means±S.E.M. and were analyzed by one-way ANOVA followed by a Fisher's *post-hoc* multiple comparison test.
