# Supplementary figures and images for: Autophagy fails to prevent glucose deprivation/glucose reintroduction-induced neuronal death due to calpain-mediated lysosomal dysfunction in cortical neurons
Source: Cell Death Dis. 2017 Jun 29;8(6):e2911–. doi: 10.1038/cddis.2017.299 (PMC5520945; doi:10.1038/cddis.2017.299)

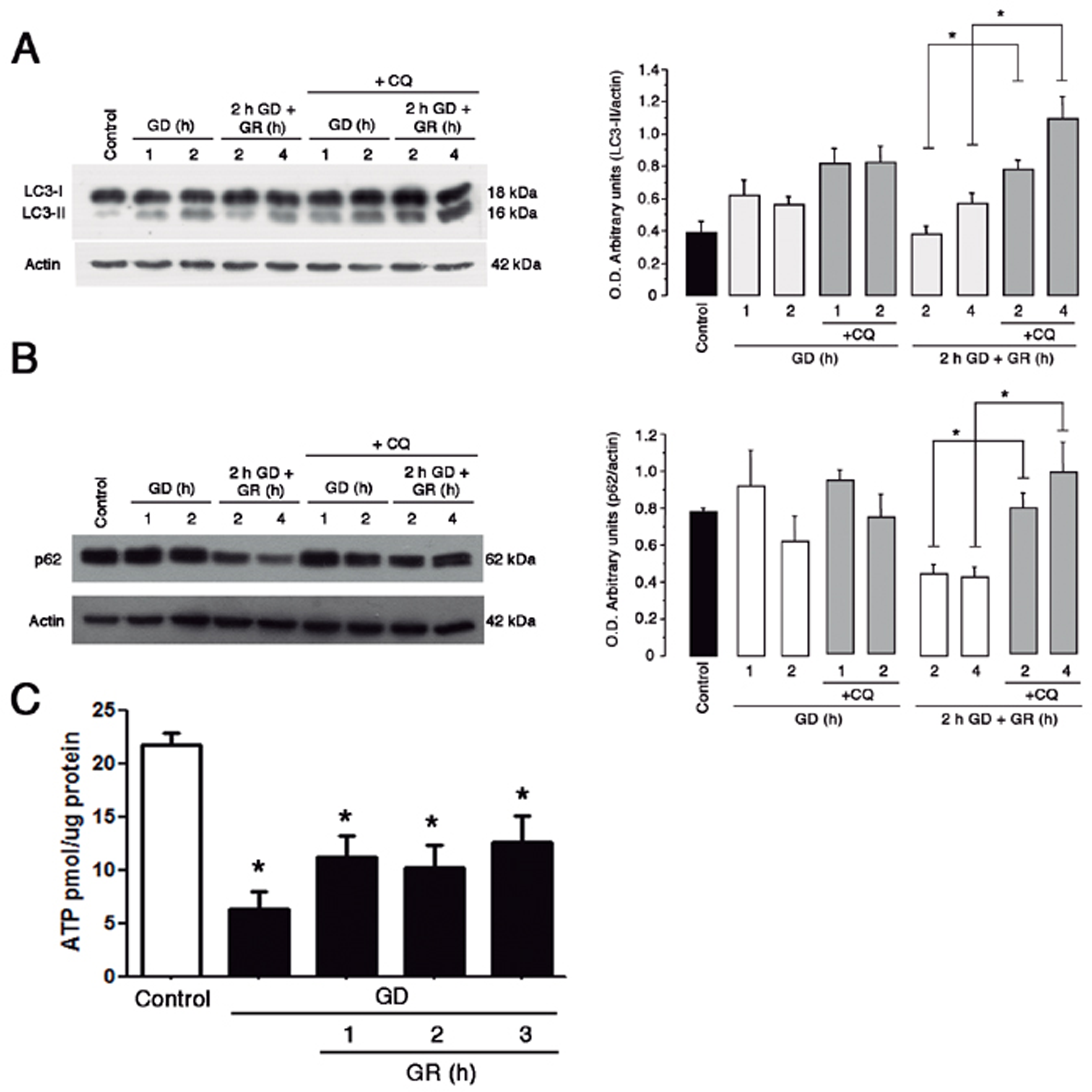

Supplement: Supplementary Figure S1 [file cddis2017299x1.tif]

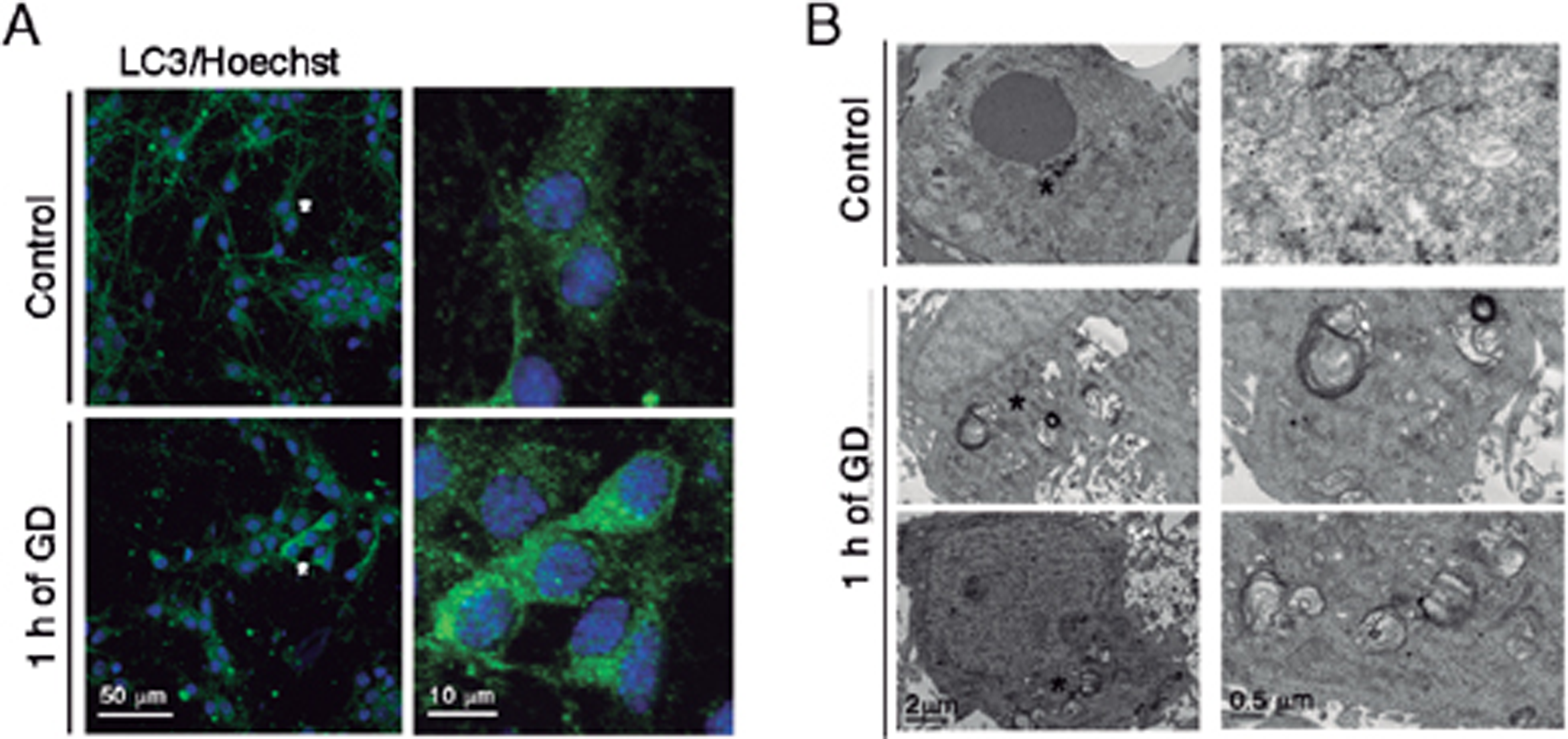

Supplement: Supplementary Figure S2 [file cddis2017299x2.tif]

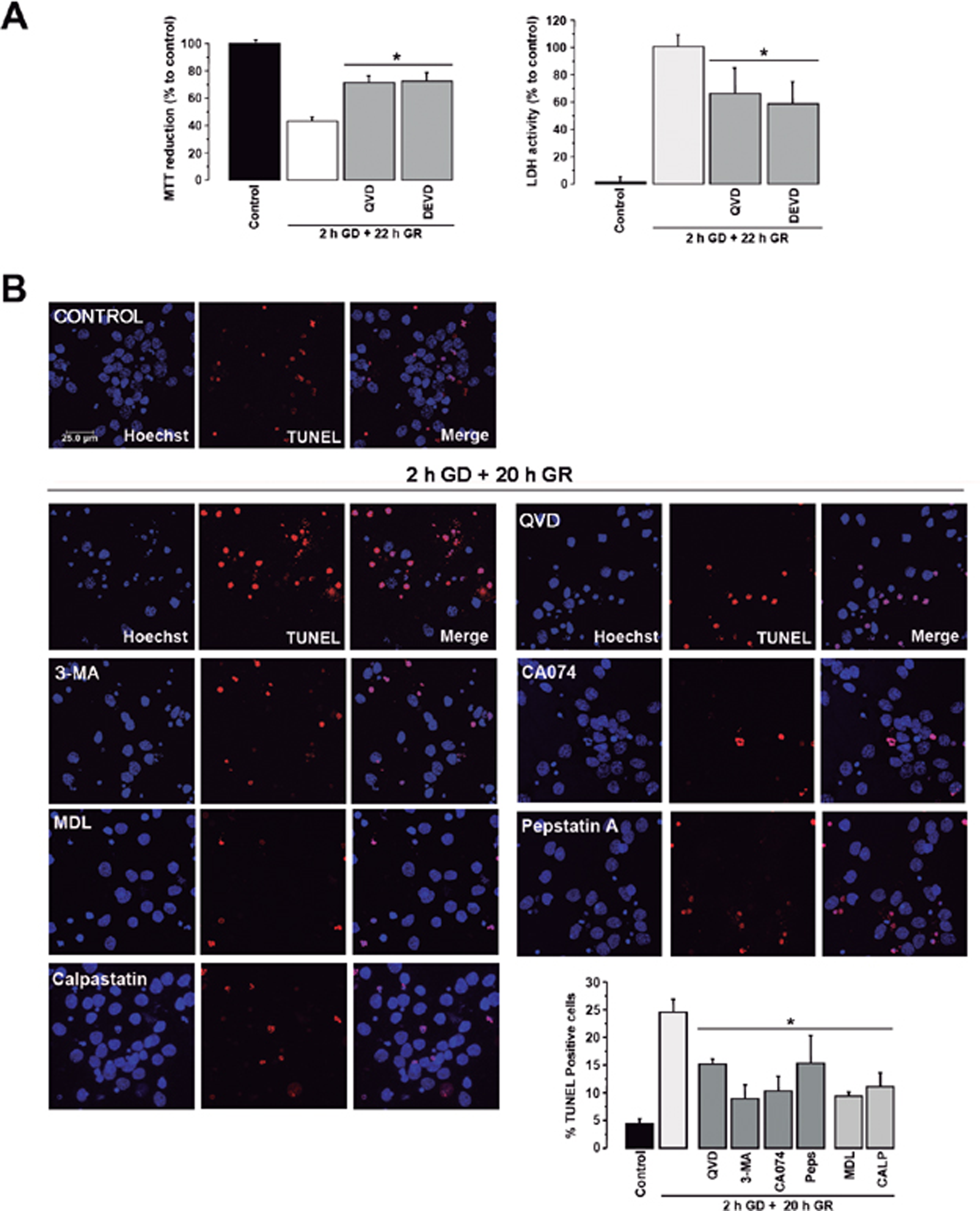

Supplement: Supplementary Figure S3 [file cddis2017299x3.tif]

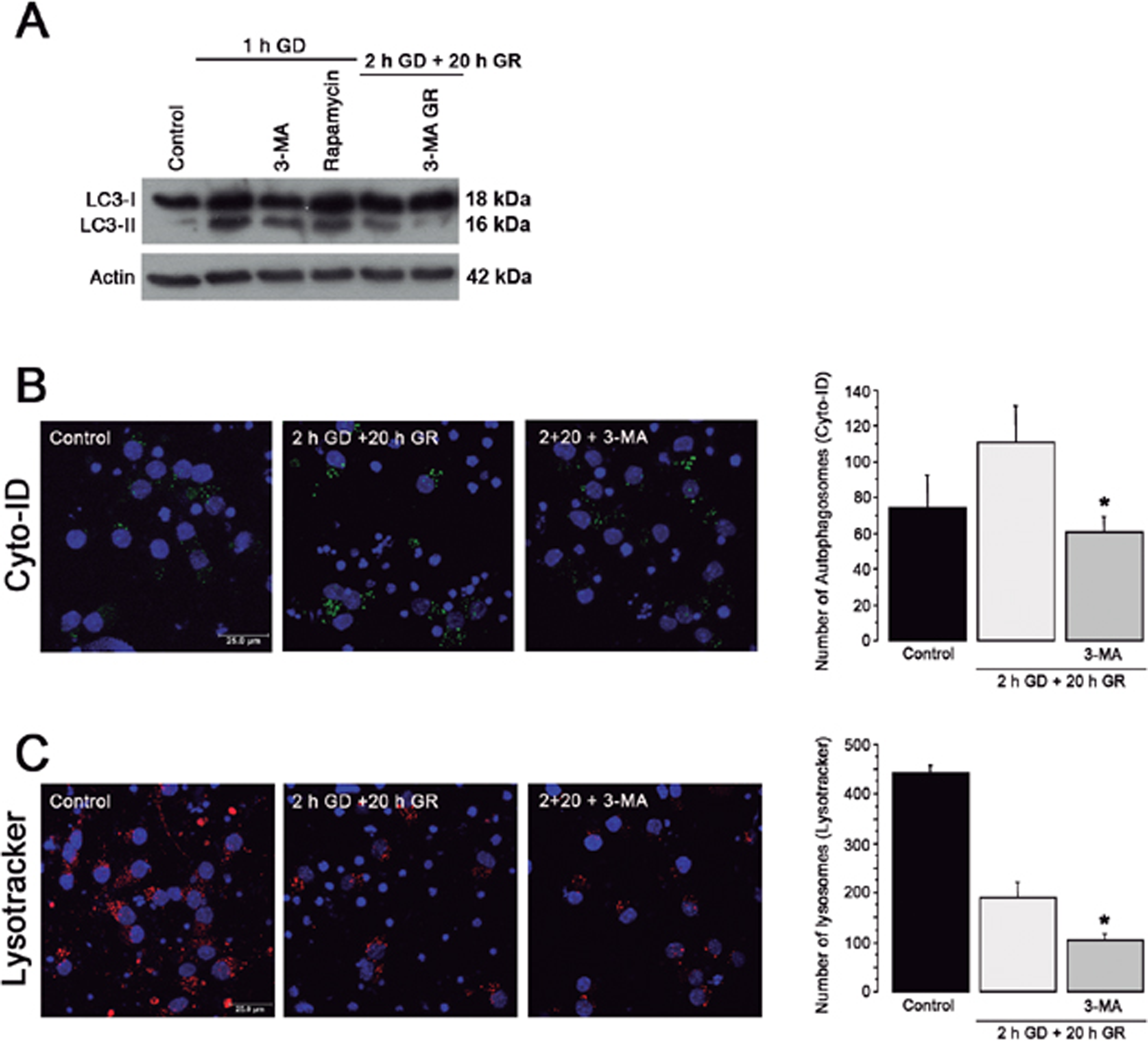

Supplement: Supplementary Figure S4 [file cddis2017299x4.tif]

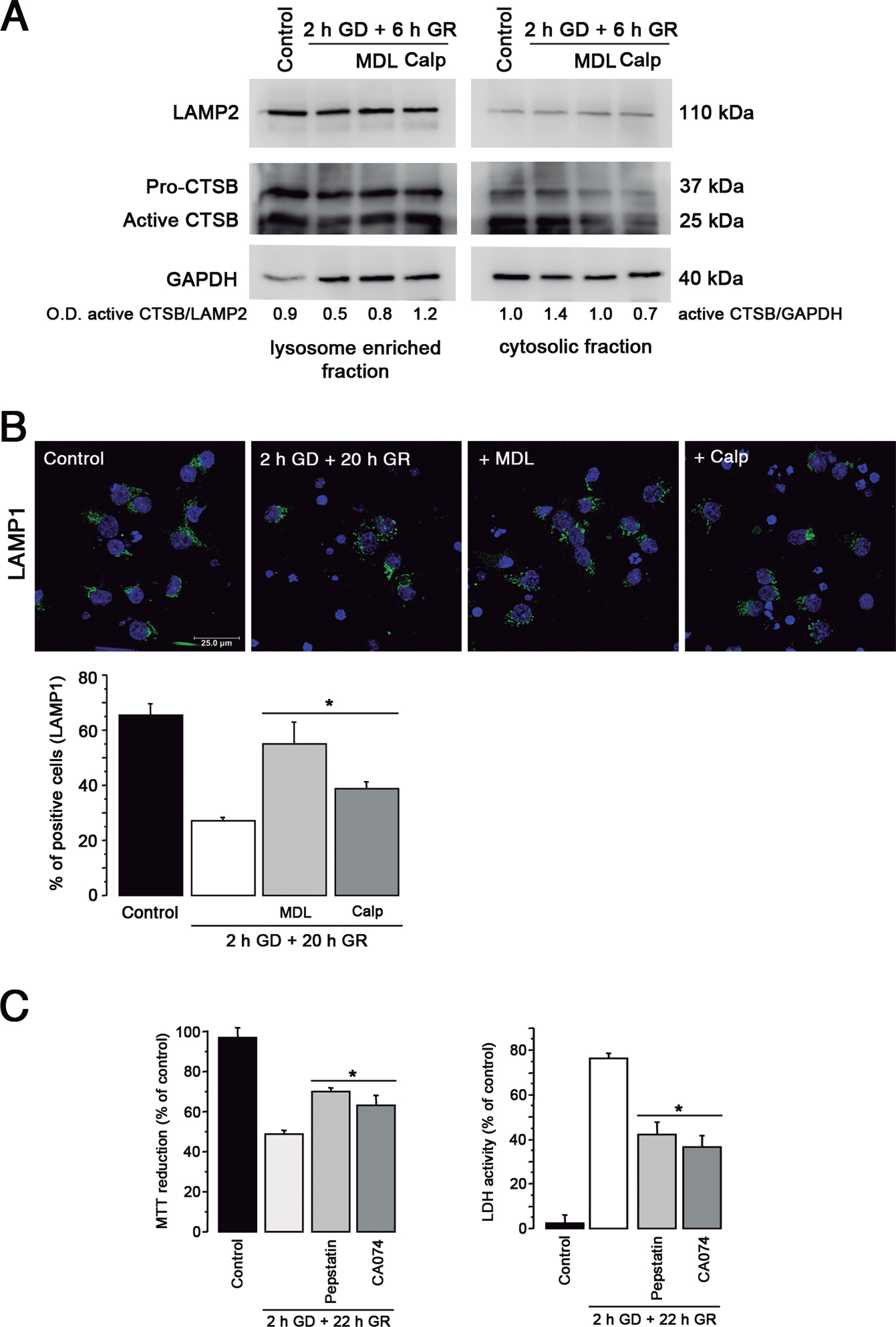

Supplement: Supplementary Figure S5 [file cddis2017299x5.tif]
