# Supplementary material for: Autophagy fails to prevent glucose deprivation/glucose reintroduction-induced neuronal death due to calpain-mediated lysosomal dysfunction in cortical neurons
Source: Cell Death Dis. 2017 Jun 29;8(6):e2911–. doi: 10.1038/cddis.2017.299 (PMC5520945; doi:10.1038/cddis.2017.299)
Supplement: Supplementary Figure Legends [file cddis2017299x6.docx]

**Supplementary Figure Legends**

Supplementary Fig 1. The autophagic flux was blocked during GD and reestablished during GR. Representative immunoblots and quantification of LC3-II/Actin ratio (A) and p62/SQSTM1/Actin ratio (B) after 1-2 h GD and 2-4 h GR in the presence or absence of CQ. ATP concentration after GD/GR (C). Bars represent mean ± SEM (n=4-5). Data were analyzed by One way ANOVA followed Fisher’s post hoc test *p<0.05 vs GD without CQ (A-B) or vs control (C).

Supplementary Fig 2. GD induces the formation of autophagosomes. LC3 immunocytochemistry in control cells and cells exposed to 1h GD (A). Electron microscopy images of control cells and cells exposed to 1 h GD showing double and multi-membrane vesicle-like structures (B).

Supplementary Fig 3. Caspase inhibition reduces neuronal death. MTT reduction and LDH activity of cortical cultures exposed to GD/GR in the presence or the absence of caspase inhibitors (A). Images showing nuclei stained with TUNEL (red) and Hoechst (blue) of cortical neurons exposed to GD/GR in the presence or absence of 3-MA, MDL, Calpastatin, QVD, CA074 or pepstatin. Inhibitors were added during GR. Graph shows the number of TUNEL-stained nuclei in the presence of the absence of the different inhibitors (B). Bars represent mean ± SEM (n=5-7). Data were analyzed by One way ANOVA followed Fisher’s post hoc test *p<0.05 vs GD.

Supplementary Fig. 4. Autophagy inhibition by 3-MA reduces autophagosome and autolysosome/lysosome formation. Representative immunoblot showing the effect of 3-MA added during GD (lane 3) or GR (lane 6) (A). Representative confocal images showing autophagosomes (B) and lysosomes (C) in the different experimental conditions and their quantification. Bars represent mean ± SEM (n=4-7, B and 3-4 C). Data were analyzed by One way ANOVA followed Fisher’s post hoc test *p<0.05 vs GD.

Supplementary Fig. 5 Calpain inhibition prevents LAMP1 immunoreactivity reduction and CTSB extralysosomal leakage and CTSB inhibitors increase cell survival. Representative immunoblot showing pro-CSTB (37 KDa) and active CTSB (25 KDa) in the lysosome enriched and cytosolic fractions in the absence or the presence of calpain inhibitors (A). Confocal images showing the effect of calpain inhibitors on LAMP1 immunoreactivity and its quantification (B). Effect of cathepsin inhibitors on cell viability (C). Inhibitors were added during GR. Bars represent mean ± SEM (n=4, A and 3-4 C). Data were analyzed by One way ANOVA followed Fisher’s post hoc test *p<0.05 vs GD.
